# Analyses of Hybrid Viability across a Hybrid Zone between Two *Alnus* Species Using Microsatellites and cpDNA Markers

**DOI:** 10.3390/genes11070770

**Published:** 2020-07-09

**Authors:** Jan Šmíd, Jan Douda, Karol Krak, Bohumil Mandák

**Affiliations:** 1 Faculty of Environmental Sciences, Czech University of Life Sciences Prague, Kamýcká 129, 165 00 Praha-Suchdol, Czech Republic; smidjan@fzp.czu.cz (J.Š.); douda@fzp.czu.cz (J.D.); krak@fzp.czu.cz (K.K.); 2 Institute of Botany, Czech Academy of Sciences, Zámek 1, CZ-252 43 Průhonice, Czech Republic

**Keywords:** germination, chloroplast DNA, introgression, microsatellites, polyploidy, tension zone

## Abstract

Diploid *Alnus glutinosa* s. str. and autotetraploid *A. rohlenae* form a narrow hybrid zone in a study area in southern Serbia, which results in triploid hybrid formation. The vast majority of previous studies have been focused on studies of maternal plants, but the offspring resulting from their crossing have not been much studied. Here, we use the variability of microsatellites and chloroplast DNA between these species and their putative hybrids to create an overall picture of the development of the hybrid zone and its predicted type. To elucidate the gene transfer within both species, the origins of individual ploidies and especially the role of triploid hybrids, a germination experiment was carried out linked with a flow cytometry study of the resulting seedlings. The tension zone model seems to offer the most adequate explanation of our observations, with selection against triploid hybrids and the spatial positioning of the hybrid zone. Despite selection against them, the triploid hybrids play an important role in the exchange of genes between the two species and therefore serve as a bridge for introgression. The presence of fertile triploids is essential for enriching the haplotype diversity between these species and for the development of new genetic lineages.

## 1. Introduction

It is generally accepted that most hybrid zones are maintained in a balance between naturally occurring selection and the diffusion of genes by dispersal across the boundary species [1,2]. Three main types of hybrid zone are recognised. These are (1) a bounded hybrid superiority zone, (2) a tension zone and (3) a mosaic hybrid zone. The type of hybrid zone formed depends mainly on hybrid fitness, selection against them and gene flow between the parents and the hybrids [1,3]. In a bounded hybrid superiority zone, the hybrids have greater fitness and they occupy the habitat intermediate between the habitats of the two parents. In a mosaic hybrid zone, overlap can be extensive, and occupied habitat patches can appear and disappear with shifting species distributions. In the tension zone model, the hybrid zone is maintained by selection against individuals of mixed ancestry and the hybrid zone is also able to move [1]. Current research focuses on identifying the mechanisms that limit hybrid formation and/or reduce the fitness of the resulting hybrids. Nevertheless, the mechanisms that maintain species barriers in many hybrid zones can be much more complicated than in these three abovementioned examples [4,5,6,7,8]. Some hybrid lines can have lower fitness due to genetic complications, but hybrid fitness is usually inconstant and thus highly variable, and such populations often contain some relatively fit and fertile genotypes with the potential to contribute new genetic variation to future generations [4,9,10]. Parental species can also coexist with highly fit hybrids without being absorbed if selection favours the hybrids, because favourable epistatic gene combinations are not always heritable between generations, and the higher fitness of the hybrids may be limited to the area of the hybrid zone [2,6,7,11].

Environmental context and species life history traits are often essential components of hybrid success. Many plant species can tolerate a wide range of environmental conditions at the adult stage but may require a much narrower range of conditions for effective seed germination and seedling establishment [4,12]. Closely related species or even individual genetic lineages within a species may express very contrasting requirements for germination [13,14]. If the conditions for germination are highly specific, genetic changes resulting from hybridisation may affect the “regeneration niche” and drive the ecological divergence of hybrid populations [10,14].

The phylogeographic history of *Alnus glutinosa* s. str. “black alder” has been studied extensively. Like many other temperate species, black alder survived the last Ice Age in multiple refugia in the Iberian, Apennine and Balkan Peninsulas [15,16]. These refugia served for the recolonisation of the northern regions of Europe. In central and northern Europe, colonising lineages formed secondary contact zones when they met a number of times during the colonisation period. However, it has been found only quite recently that in the Iberian and Balkan Peninsula, in addition to the diploid black alder, a tetraploid cytotype also exists [17]. In contrast to the diploid populations of *A*. *glutinosa* s. str., the tetraploids never participated in the colonisation of northern Europe. Their morphological distinctiveness, allopatric distribution and genetic differentiation has led to the description of two new species of tetraploid *Alnus* cytotypes by Vít et al. [18]. While in the Iberian Peninsula, the tetraploid cytotype was described as *Alnus lusitanica* Vít, Douda and Mandák, in the western Balkan Peninsula, we find another tetraploid species, *Alnus rohlenae* Vít, Douda and Mandák. Both are of autotetraploid origin and give no indication that the closely related *A. incana* has been involved in their evolutionary history [17]. The distribution range of the autotetraploid *A. rohlenae* (2n = 4x = 56) lies in the mountainous part of the western Balkan Peninsula [17,18]. This species is found to be an endemic to this area and differs from the diploid *A. glutinosa* s. str. (2n = 2x = 28) both morphologically and also genetically [18]. The morphological differences between the two species decreases when they occur in proximity to one another. Šmíd et al. [19] found that *A. rohlenae* commonly hybridises with *A. glutinosa* s. str., and their triploid hybrid is likely responsible for gene exchange between the two species. Mandák et al. [17] and Šmíd et al. [19] discovered several areas with mixed ploidy populations. These areas were found in Bosnia and Herzegovina in the Neretva River canyon and in the northern part of the country on the Spreča River, in southern Serbia and in northern Greece. In our previous study, we discovered a well-defined hybrid zone in southern Serbia. In this area, the hybrid zone is quite large due to the gradual descent from the hills of the Dinaric Mountains to the Pannonian plain.

In this study, we focus on the previously discovered hybrid zone between the diploid *A. glutinosa* s. str. and its autotetraploid *A. rohlenae* in Serbia (see [17,18]). We analysed the evolutionary processes operating in the hybrid zone using several approaches. (1) We used flow cytometry to determine the frequency of the occurrence of diploid, triploid and tetraploid taxa. (2) Using cpDNA variation, we determined the direction of the hybridisation process, as cpDNA is maternally inherited in most angiosperms [20], and we also reconstructed a phylogenetic tree of the two species and their putative hybrid within the study area. (3) Using analyses of microsatellite variation, we determined the genetic composition of individual species. (4) To estimate the fitness of each ploidy, we tested each ploidy level in a germination experiment. (5) To evaluate whether the triploid individuals originated solely due to hybridisation between diploids and tetraploids, we analysed the ploidy level in all the seedlings obtained in a germination experiment.

We hypothesise that the low presence of triploid hybrids detected in previous studies [17,19] may point to the operation of a tension hybrid zone model, based on selection against hybrids. Specifically, we asked (i) “What is the type of hybrid zone?”, (ii) “Are there any differences in germination between the diploids and polyploids?”, (iii) “Do the seedlings of diploids and tetraploids have the same ploidy as their parents?”, and (iv) “Are the triploids sterile or not, and what is the ploidy level composition of the offspring?”.

## 2. Materials and Methods

### 2.1. Study Site and Sample Collection

**Figure 1:**
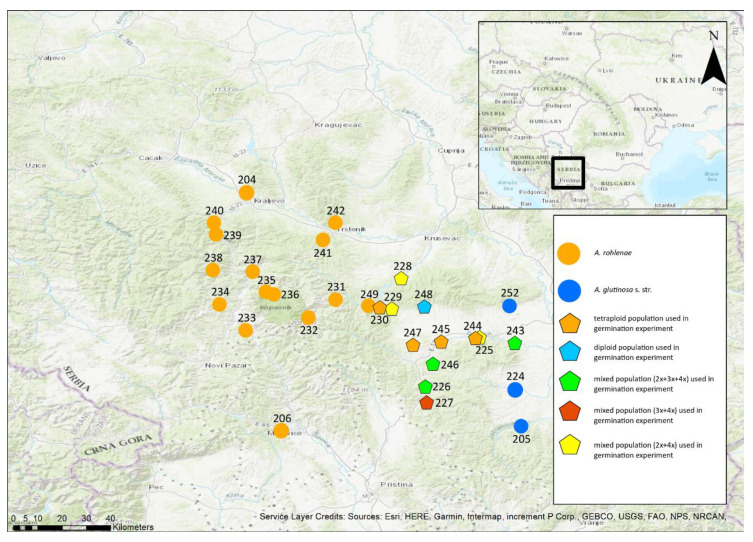
Geographical locations of populations of *Alnus glutinosa* s. str., *A. rohleane* and their triploid hybrid across the entire study area. Numbers of the populations are provided in the figure.

The research area is located in southern Serbia and bordered by the river South Morava to the east, the river Western Morava to the north, the river Ibar to the west and the border with Kosovo to the south (Figure 1). This study region contains both lowlands and the Kopaonik Mountains, which are considered the natural border between the two species. Because the exact boundaries of the hybrid zone were uncertain, we sampled from as many rivers as possible within the study region. The sampling was performed in two phases. During July 2018, a first sampling involved collecting two to five individuals per population to find the approximate position of the hybrid zone. After the analysis of this preliminary dataset, the second sampling was carried out in October 2018, when 20 samples (wherever possible) were collected per population. All the sampled trees were marked for future identification during an autumn collection of fruits. In total, 287 individuals from 30 populations were analysed by flow cytometric analysis (FCM) to estimate the ploidy level (Figure 1). To analyse chloroplast DNA, 234 individuals from 29 populations were used, and to analyse microsatellites, 204 individuals from 26 populations were used.

In October of 2018, the fruits of *A. rohlenae* and *A. glutinosa* s. str. were collected. The fruits ripen from late September, and the cones persist on the branches until the end of winter. Across the hybrid zones, we collected only fully ripe cones. The collected cones were stored at room temperature in paper bags for four months to dry out. In total, we collected seeds from 66 individuals from 11 populations (Supplementary Table S1). Four of the individuals were triploids, nine were diploids and 53 were tetraploids.

### 2.2. Cytotype Screening

Cytotype screening was done using fresh leaves of *A. glutinosa* s. str. and *A. rohlenae* collected from natural populations in the field. The leaves were placed in plastic bags and held in a refrigerator (~5 °C) pending FCM analysis. Herbarium vouchers were collected from all the populations (three vouchers per population) and deposited at the Faculty of Environmental Sciences (CULS).

In total, 30 populations and 287 individuals were analysed for ploidy level using FCM. Nuclei were released by chopping up approximately 0.5–1 cm^2^ of fresh leaf together with the same amount of the leaves of common daisy (*Bellis perennis* L.; 2C = 3.38 pg; [21]) as a standard plant. Leaves were homogenised in Petri dishes, each containing 0.5 mL of Otto Ι buffer (0.1 M citric acid and 0.5% Tween-20; [22]). The nuclear suspension was filtered using a fine nylon mesh and incubated for about 5 min at room temperature. Finally, 1 mL of staining Otto II solution (0.4 M Na_2_HPO_4_ × 12 H_2_O, 2 μL/mL of β-mercaptoethanol and 4 μg/mL of DAPI) was added, and the samples were measured using a Partec Space cytometer (Partec GmbH, Munster, Germany) equipped with a 365 nm UV-LED as a source of UV light for DAPI excitation.

### 2.3. Chloroplast DNA (cpDNA)

The spacers *ndh*F-*rpl*32, *psb*J-*pet*A and *3′rps*16-*5′trn*K [23] were chosen and sequenced based on our previous studies (see [16,17]) The PCRs were carried out in 15 µL volumes, each containing 7.5 µL of Qiagen Multiplex PCR kit (Qiagen, Germany), 0.5 mM concentrations of each primer, 5.5 µL of nuclease free water and 1 µL of DNA (20–25 ng/µL) The cycling conditions were 95 °C for 15 min followed by 40 cycles of 95 °C for 30 s, 49 °C (*ndh*F-*rpl*32, *psb*J-*pet*A) or 52 °C (3′*rps*16-5′*trn*K) for 30 s and 72 °C for 2 min. The reactions were completed by a final elongation step at 72 °C for 10 min. The PCR products were checked on 1.5% agarose gel and sent to Macrogen (Amsterdam, Netherlands) for sequencing. The sequencing of both DNA strands was carried out using the original PCR primers.

### 2.4. Microsatellite Analysis (SSRs)

The genetic variation was derived from 19 pairs of microsatellite primers that were composed into two multiplexes. The primers for the first multiplex (M1: A2, A7, A10, A22, A26, A35, A37 and A38) were chosen following [24], and the primers for the second multiplex (M2: AG1, AG5, AG9, AG10, AG13, AG20, AG23, AG25, AG27, AG30 and AG35), following [25].

DNA was amplified using the Quiagen Multiplex PCR kit in a total reaction volume of 5 µL of PCR mix. The composition of the PCR mix was 1 µL of DNA, 0.025–0.3 µL of each primer and 3 µL of Master Mix (Quiagen). The PCR amplifications were conducted in a Veriti Thermal Cycler (Applied Biosystems, USA) under the following conditions for M1: 15 min of denaturation at 95 °C, followed by 40 cycles at 94 °C for 30 s, 30 s at 58 °C, 1 min at 72 °C and a final extension of 10 min at 72 °C; and for M2: 5 min denaturation at 95 °C, 30 cycles of 95 °C for 30 s, 58 °C for 3 min and 72 °C for 30 s, and an extension of 30 min at 60 °C. A 1 µL aliquot of PCR product was mixed with 0.2 µL of GeneTrace-500 LIZ (Carolina Biosystems) and 12 µL of Hi–Di formamide (Applied Biosystems), and the fragments were separated by capillary electrophoresis using a ABI 3500 genetic Analyser. Allele sizes were determined using GeneMarker version 2.4.0 (SoftGenetics, USA). A microsatellite locus was treated as missing data after two or more amplification failures.

### 2.5. Germination Experiment

All the seeds from tetraploids were combined within each population, as were all the seeds from diploids. The seeds from triploids were germinated as individuals due to the low numbers of triploids collected. This provided a group of nine tetraploid populations, three diploid populations and four triploid individuals.

Six to eight replicates of ten seeds each were germinated under one or another of two different temperature regimes on filter paper (90 mm in diameter) in a Petri dish. The warmer temperature regime (“warm treatment”) was selected based on the maximum and minimum temperatures in April for the lowlands in our study area with diploid predominance. The cooler temperature regime (“cold treatment”) was selected for the mountainous area with tetraploid predominance. For both climatic regimes in this study, a 21-day pre-chill was necessary for successful germination [26]. The seeds were stratified at 5 °C in Petri dishes filled up with distilled water. After stratification, the “warm treatment” was set at 25/15 °C (day/night) with 16 h of light and 8 h of dark. The “cold treatment” was set at 20/10 °C (day/night) with 16 h of light and 8 h of dark. Germination was checked every 48 h, when all the germinated seeds and seedlings were immediately removed and transferred to a growth chamber with a single temperature regime of 20/15 °C (day/night) with 16 h of light, 8 h of dark and 70% humidity.

### 2.6. Statistical Analyses

#### 2.6.1. Chloroplast DNA (cpDNA)

Sequences were aligned manually using BioEdit 7.0.5 [27] and then combined with FaBox [28]. Mononucleotide repeats were excluded due to a potentially high level of homoplasy [29] before processing the data further. Indels were coded by the simple gap coding method [30] as implemented in SeqState 1.4.1 [31]. Heuristic searches for most parsimonious trees were carried out with PAUP 4.0.b10 [32], and Bayesian phylogenetic inference was carried out using MrBayes 2.3.2. [33]. MrModeltest 2.3 [34] determined the most plausible test for Bayesian analysis. Each haplotype was represented by one sequence, and, as the outgroup, three haplotypes of *Alnus viridis* (see [16]) were included in the dataset. The data were then divided into two sets (nucleotide sequence and presence/absence matrix for coded indels). In MrBayes, the analysis was computed for 5 million generations. After the run, the average standard deviation was lower than 0.01; therefore, the convergence was reached. After 20% burn-in, the remaining trees were used to construct a consensus tree. A posterior sample of trees was calculated using BEAST 2.1 [35]. Three independent runs with the same parameters were carried out and combined into a single dataset using LogCombiner [36]. The BEAST was run for 500 million Markov chain Monte Carlo (MCMC) chain lengths (sampled every 2000 generations; burn-in, 25%). The SYM+G model was deemed the most suitable for our dataset according to the Akaike information criterion and was set up as a GTR model with frequencies “all equal” and a gamma category count of “4”, assuming coalescent constant population priors with a relaxed lognormal clock and clock rate, 1.1 × 10^−9^ [37]. The convergence for all the parameters was assessed using Tracer 1.7 [38].

#### 2.6.2. Microsatellite Analyses

To determine the level of genetic diversity of *A. glutinosa* s. str. and *A. rohlenae*, the number of alleles (*A*), Nei’s [39] gene diversity (*He*) and the coefficient of inbreeding (*Fis*) were calculated using the SPAGedi 1.5 [40] software. The Bayesian model-based clustering of microsatellite data was carried out in STRUCTURE 2.3.3 [41]. Due to the presence of mixed ploidy populations, the data were transformed to single ploidy (tetraploids) in POLYSAT 1.3-0 [42]. The R software [43] using the POLYSAT package generated the input data file for STRUCTURE computations. Ten replicates for each K = 1–27 (the user-defined number of clusters) with a burn-in length of 100,000 generations and the data collection of the additional 1,000,000 generations were run (Markov chain Monte Carlo), using the admixture model and correlated allele frequencies. The STRUCTURE output data were parsed using the Structure Harvester [44] to determine the optimal K value [45] and to obtain the input file for the cluster assignments then conducted in CLUMPP 1.1.2 [46]. The bar plot was visualised using DISTRUCT 1.1 [47]. Principal coordinate analysis (PCoA) was performed using POLYSAT to examine genetic similarities and relationships among the individuals of the three cytotypes.

#### 2.6.3. Origin of Hybrids

NEWHYBRIDS 1.1 [48]-estimated Bayesian posterior probabilities were generated by a Markov chain Monte Carlo method for the purpose of classifying hybrids into six genotype frequency classes—in our case, pure *A. glutinosa* s. str., pure *A. rohlenae*, F1 hybrids, F2 hybrids, F1 hybrid backcrosses to *A. glutinosa* s. str. (AR Bx) and F1 hybrid backcrosses to *A. rohlenae* (AG Bx). The microsatellite data were transferred to a binary matrix based on the presence/absence of particular alleles. One pure diploid (248) and one pure tetraploid (233) population were chosen as the outgroup for prior information (“z option”) to determine in advance to which genotype frequency category they belonged. Then, ten independent runs of 1,000,000 iterations after a burn-in of 100,000 iterations were used.

#### 2.6.4. Germination

The effects of population, species and treatment on the final germination percentage and rate of germination were analysed using analysis of variance (ANOVA). The final germination percentage corresponded to the proportion of seeds germinating during 24 days of temperature treatment. The arcsin square root transformation of the final percentage germination for the seeds was used to meet the assumptions of ANOVA. The time (days) at which 50% of the seeds had germinated was used to express the rate of germination (*t50*). Multiple comparisons were made using Tukey’s post-hoc tests using the function glht in the package multcomp [49]. All the analyses were carried out using R.

## 3. Results

### 3.1. Cytotype Composition of Adult Trees

Of the 30 populations, tetraploids occurred in 74.9% (215 individuals); triploids, in 3.5% (10 individuals); and diploids, in 21.9% (63 individuals) (Figure 1). Three populations contained all three ploidies, four populations contained two ploidies, and the remaining 23 populations contained only one ploidy. Among the populations that contained a single ploidy, 19 populations contained tetraploids and four populations contained diploids. The average sample-to-standard ratios (Index) of the samples analysed ± SD were for the diploids, 0.306 ± 0.004; for the triploids, 0.472 ± 0.006; and for the tetraploids, 0.613 ± 0.025. The coefficients of variation (CV) were for the diploids, 4.15% ± 0.8 SD; for the triploids, 3.54% ± 0.84 SD; and for the tetraploids, 2.90% ± 0.62 SD. Our values of Index and CV are similar to those in the literature.

### 3.2. Chloroplast DNA Diversity

**Figure 2:**
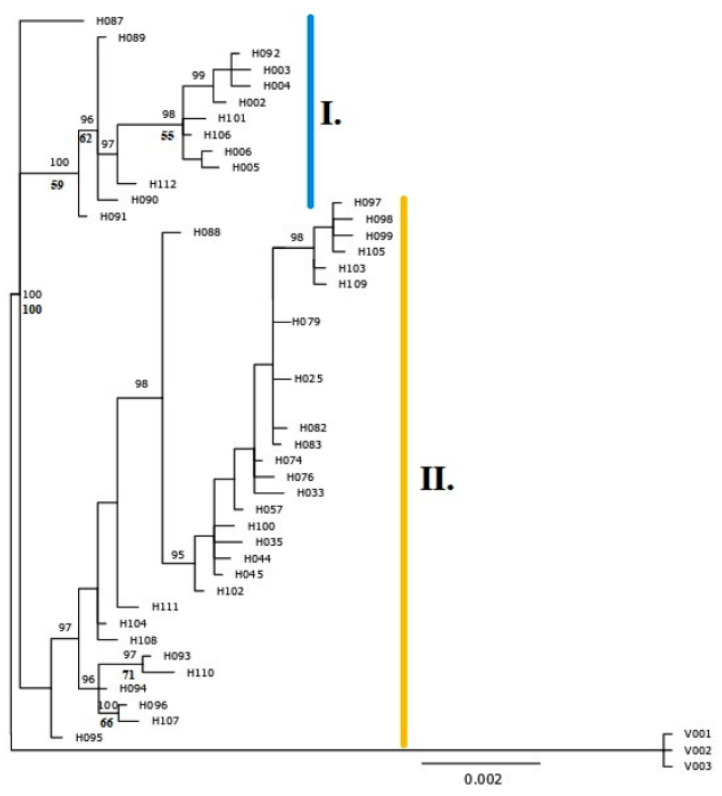
Phylogenetic relationships within the *Alnus glutinosa* s. l. in the study area estimated based on the concatenated dataset of three cpDNA spacers. Fifty per cent majority-rule consensus tree of the Bayesian phylogenetic analysis; numbers above branches indicate Bayesian posterior probabilities (in percentages; only values higher than 95% are shown), and numbers below branches, bootstrap support (in percentages; only values higher than 50% are shown) from the maximum parsimony analysis. All cpDNA halotypes were deposited in GenBank under Accession MT729389– MT729646. The *A. viridis* outgroup is marked as V001–V003.

We discovered 31 new haplotypes. The new haplotypes were divided into two main groups (Figure 2, Supplementary Figure S1). Haplogroup I represent most of the diploid individuals of *A*. *glutinosa* and also tetraploids from the vicinity of the hybrid zone, while Haplogroup II is mainly typical for tetraploid individuals, classified as *A. rohlenae*. Beside diploids, Haplogroup I was also present in the majority of triploid individuals, i.e., the maternal plant of these triploids was diploid *A. glutinosa* s. str. Haplogroup II was also present in two diploid individuals.

### 3.3. Microsatellite Variation

For *A. glutinosa* s. str. and *A. rohlenae*, the overall mean number of alleles per locus (*A*) was 4.78, and for gene diversity (*He*), it was 0.72 for all the populations analysed (see Supplement Table S1 for individual populations). The coefficient of inbreeding (*Fis*) in the diploid populations reached values of 0.42, and in the tetraploid populations, values of 0.33.

**Figure 3:**
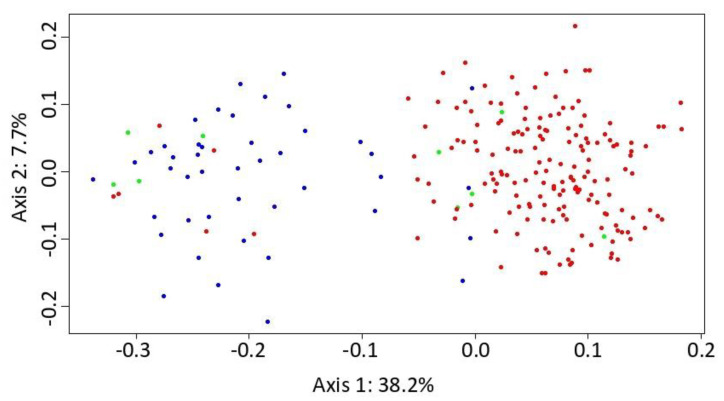
Principal coordinate analysis (PCoA) carried out in the POLYSAT package in R for *Alnus glutinosa* s. str. (blue marks), *A*. *rohlenae* (red mark) and putative triploid hybrid (green mark). The percentage of variance explained by each axis is provided in the figure.

The PCoA analysis separated *A. glutinosa* s. str. and *A. rohlenae* as two almost-distinct species. A few tetraploid individuals were assigned to the diploid group, and similarly, some of the diploid individuals, to the tetraploid group. These individuals are likely the result of gene exchange via a “triploid bridge” (Figure 3). The first component axis explained 38.2% of the total variance, and the second component axis explained a further 7.7%.

**Figure 4:**
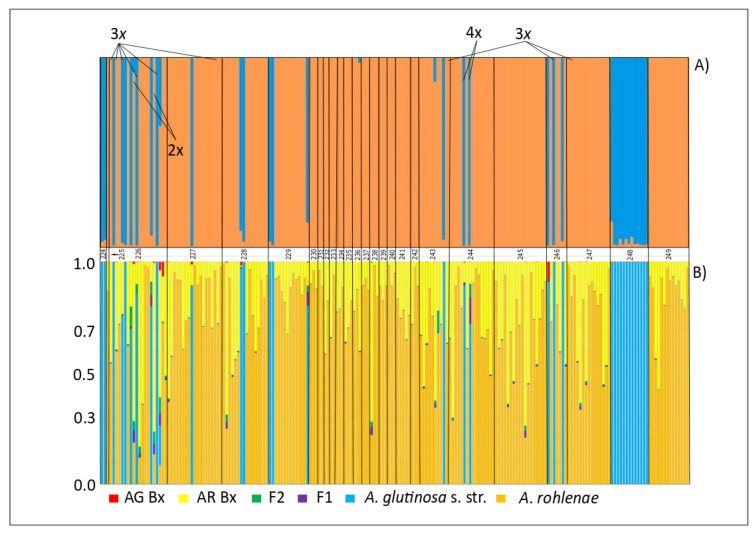
(**A**) Percentage assignment of *Alnus* individuals (represented by vertical bars) to each of the clusters determined (represented by different colours) inferred by STRUCTURE. Site codes (see Supplementary Table S1) indicate the geographical location of the populations along the x-axis. Blue colour represents the majority of the diploid and triploid individuals, and orange colour, the majority of the tetraploid individuals. (**B**) Hybrid class assignments, F1 hybrids (F1), F2 hybrids (F2), F1 backcrosses to *A. glutinosa* s. str. (AG Bx) and F1 backcrosses to *A. rohlenae* (AR Bx) from NEWHYBRIDS are plotted in line with the STRUCTURE results.

The STRUCTURE analysis provided us with results very similar to those from the PCoA analysis. Two clusters (*K* = 2) best explained the population structure following ploidy level (Figure 4). However, there were some irregularities. (1) Two tetraploid individuals with the “diploid” haplotype H092 (Haplogroup I) were assigned to the tetraploid cluster. This may indicate a fusion of unreduced diploid and reduced tetraploid gametes. (2) Two diploid individuals with the “diploid” haplotypes H089 and H091 (Haplogroup I) were assigned to the tetraploid cluster. The origins of these individuals is probably more complicated, theoretically involving a reduced haploid gamete from a triploid individual fused with a reduced diploid gamete.

Surprisingly, the triploid hybrids were assigned to the diploid or tetraploid groups by STRUCTURE without any signs of admixture. While the assignment of the three triploid individuals with “diploid” haplotypes to the diploid cluster may be a result of the fusion of reduced and unreduced gametes from diploid individuals, the assignment of two triploid individuals with “diploid” haplotypes to the tetraploid cluster is probably the outcome of hybridisation between diploid and tetraploid individuals with a diploid mother plant. One triploid individual of the “tetraploid” haplotype was assigned to the tetraploid cluster, which also indicates that a tetraploid individual could have served as a mother plant. Hence, the determination of the triploid ploidy level does not necessary mean that the individual is always the outcome of hybridisation between regular diploid and tetraploid parents.

According to the NEWHYBRIDS results, the diploids remained pure but the tetraploids did not. Most of the tetraploids were detected as *A. rohlenae* backcrosses. This seems logical if we take into account the fact that *A. rohlenae* is an autopolyploid of *A. glutinosa* s. str. The assignment of triploids is the same as in the STRUCTURE analysis (Figure 4).

To analyse the probability of the evolution of different ploidies of different origins, we further analysed the progeny of each ploidy level to determine (i) if the progeny was viable and (ii) to determine the ploidy level composition of the next generation.

### 3.4. Seed Germination

**Figure 5:**
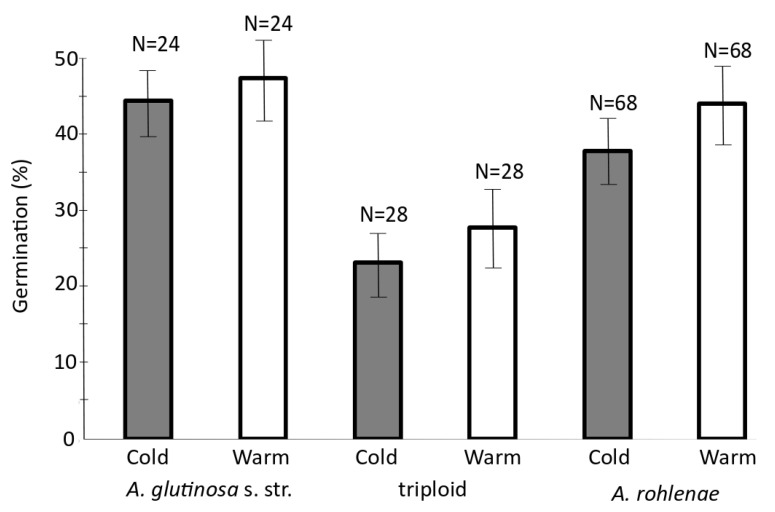
Percentages of seed germination of all three cytotypes under two climatic regimes. N = total number of repetitions with 10 seeds per repetition; error bars = Standard Error (SE). Bars bearing the same letter were not significantly different according to Tukey’s test (*p* < 0.05).

To evaluate the viability of the seeds produced by the individuals of different ploidies, we performed germination tests under two temperature regimes, each typical of conditions where diploid *A. glutinosa* s. str. and tetraploid *A. rohlenae* occur, i.e., warm and cold treatments, respectively. The final germination (%) and germination rate (t50) significantly differed between the two taxa (Table 1; Supplementary Table S2). Irrespectively of taxa, seeds germinated faster (t50) under the warm treatment. The temperature regime did not affect final germination (%) (Table 1). Generally, the diploids reached the highest germination percentage, followed by the tetraploids and then the triploids (Figure 5). In the case of the seeds collected from triploid individuals, sample 226g1228 achieved the highest germination percentage of 71.7% (43 individuals out of 60 seeds) under both temperature regimes. On the other hand, no seeds of sample 226g1240 germinated under the cold treatment, and only 1.3% (one individual of 80 seeds) germinated under the warm treatment. Surprisingly, the highest germination percentages among all the populations and ploidies occurred among the triploid plants (Supplementary Table S3).

### 3.5. Ploidy Level Variation of Seedlings

**Figure 6:**
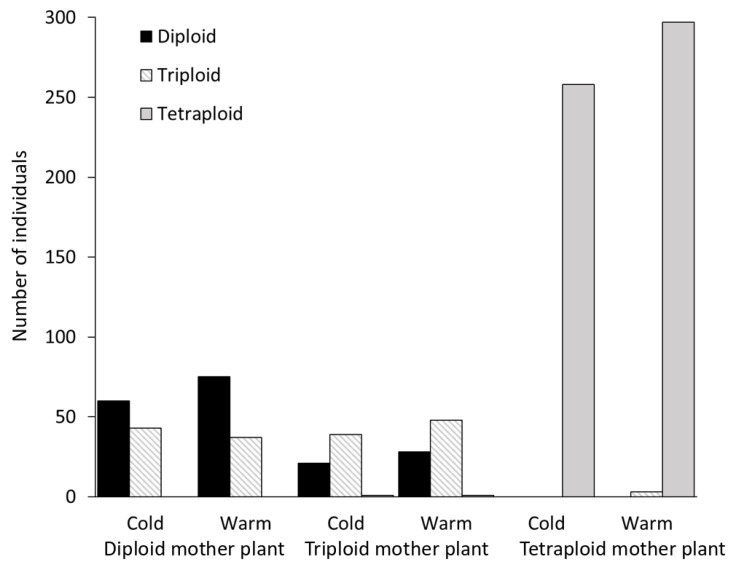
Observed ploidy levels of the seedlings and their sums in two climatic regimes (warm treatment (25/15 °C; day/night, 16/8 h) and cold treatment (20/10 °C; day/night, 16/8 h)).

Diploid and triploid individuals produced many seedlings of the same ploidy level, but with a significant number of exceptions. Besides diploid offspring, the diploid mother plants always produced a few triploid seedlings. Meanwhile, in addition to diploid and triploid seedlings, the triploid mother plants also produced a few tetraploid seedlings. Furthermore, one putatively aneuploid seedling (a ploidy level intermediate between triploid and tetraploid) germinated from the seeds of a triploid individual—sample 246g1201. Tetraploid parents almost exclusively produced tetraploid offspring, except for population 244 under the warm treatment. Here, three triploid seedlings were found (Figure 6). Therefore, we conclude that triploids can arise from both species as well as from triploids.

## 4. Discussion

**Figure 7:**
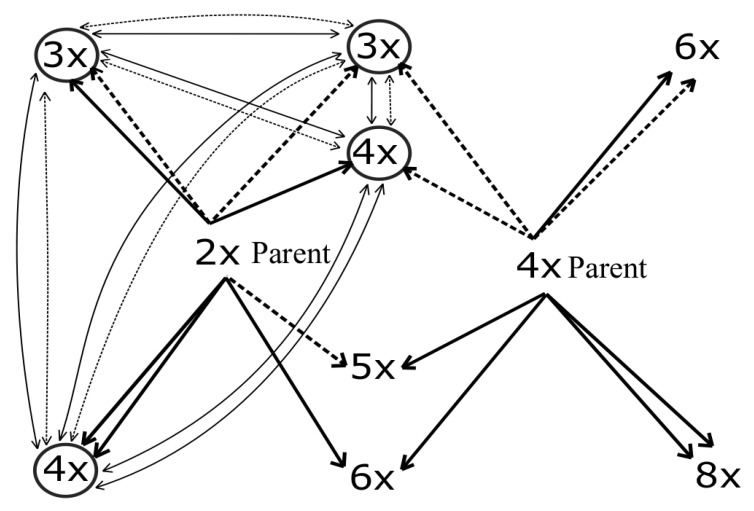
A theoretical overview of crosses involving reduced (dashed arrows) and unreduced (solid arrows) gametes of diploid and tetraploid parents (bold arrows) and possible crosses of the resulting offspring of the F1 generation (light arrows). The offspring that may arise in our case are circled.

Our study yielded a number of important outcomes concerning the establishment and maintenance of a hybrid zone lying at the margin of the distribution range of tetraploid *A. rohlenae*. Generally, the system is very variable, with many different ways in which the different ploidies can evolve. The main outcomes can be summarised as follows: (1) Several mixed ploidy populations were found, and the proportion of putative triploid hybrids is very low. (2) Both species can serve as parents, but diploid parents are more common. (3) The different ploidies can evolve along multiple paths (see Figure 7). (4) The determination of the triploid ploidy level in an individual does not necessarily mean that its evolution was by the hybridisation of diploid and tetraploid species. Instead, other processes may have played important roles, such as the fusion of reduced and unreduced gametes from one species and introgressive hybridisation. (5) In general, seeds collected from diploids exhibited the highest germination percentages, followed by seeds from tetraploids and then those from triploids. Nevertheless, some triploids do express high germination percentages. (6) Triploids may arise from both species, as well as from triploids.

### 4.1. Origin of Individual Ploidy Levels

While significant gene flow can be observed where the two species are in direct contact, away from the contact zone, the two populations exhibit no signs of hybridisation. Bayesian clustering analysis provided by the STRUCTURE software supports the division of the population into two clusters that are tightly correlated with ploidy level and species. However, some of the individuals from the mixed populations, or lying within the hybrid zone, express genetic clusters of different cytotypes. This may indicate a number of processes leading to the formation of the various ploidies.

Firstly, tetraploids may evolve by (i) the fusion of unreduced diploid and reduced tetraploid gametes. Thus, even though a plant may be tetraploid, it is likely to be closer to the diploids genetically and probably also morphologically. (ii) Furthermore, progeny analysis (Figure 6) shows that a triploid parent can produce tetraploid offspring, and other processes such as the fusion of reduced and unreduced gametes from triploids or the hybridisation of unreduced triploid and reduced diploid gametes possibly also form tetraploid plants. The formation of tetraploid individuals from diploid and triploid crosses is relatively common. Burton et al. [50] described a similar formation of tetraploids from 2x × 3x crosses in *Chamerion angustifolium* (Onagraceae). In this case, the tetraploids were formed de novo through the triploid bridge. The triploid bridge contributes 70% of the total rate of autotetraploid formation in selfing taxa, while in allopolyploids, it contributes only 1%. On the other hand, tetraploids can also be formed from 4x × 3x crosses by the union of n = 2x, which is quite common in triploids [50,51]. Ramsey and Schemske [51] state that triploids produced in diploid populations generate tetraploids via backcrossing to diploids, triploid selfing or crossing among triploids. It follows that the formation of different products of hybridisation with different levels from their parents is probably also expressed morphologically, and this complicates species determination in a hybrid zone.

Secondly, diploid individuals with “diploid” haplotypes were assigned to the tetraploid cluster. The origins of these individuals are more complex and thus harder to explain. When we know from progeny analysis (Figure 6) that many different ploidies can evolve from individuals of various parentages, there are different ways of obtaining this unusual combination. This combination was likely achieved by the introgression of cpDNA from tetraploids to diploids via triploids during crosses and backcrosses of one or multiple hybridisation events. Triploids, that serve as “bridges” for introgression, are responsible for gene exchange and unusual haplotype combinations in many other plant species [52,53,54,55]. Cases both in tetraploids and in diploids provide evidence for cpDNA introgression in the absence of the introgression of analogous SSRs. These have been described in plants in plastid or nuclear genomes with maternal or biparental modes of transmission, respectively [56,57,58]. The conflict between nuclear and chloroplast markers may already be active in the early diversification of the genus, and a common ancestor can be responsible for the chloroplast capture [58].

Thirdly, triploids are usually considered to be hybrids between diploid and tetraploid species so would not have this origin at all. We suppose that at least some of the triploids evolved along a different path, such as by the fusion of reduced and unreduced gametes from diploid individuals. Hence, these triploids have nothing to do with the “usual” hybridisation process involving the two ploidies but instead represent an autotriploid from the diploid plant. Hence, the determination of a triploid ploidy level in an individual does not necessary indicate that it is the outcome of hybridisation between regular diploid and tetraploid parents. Triploid individuals originating from the fusion of reduced and unreduced gametes probably appear everywhere in the *A. glutinosa* s. str. distribution range as has been shown by Mandák et al. [17], who found a triploid individual in an Austrian population, i.e., very far away from the distribution range of *A. rohlenae*.

The hybridisation of *Chamerion angustifolium* revealed the formation of triploids from 2x × 2x, 2x × 3x, 4x × 3x and 2x × 4x crosses, in different proportions. However, the triploids arose with least probability from 2x × 2x crosses by the fusion of unreduced gametes [50,59], while 2x × 3x and 2x × 4x crosses are generally more successful for triploid formation [51,59]; the estimated frequency of unreduced gametes in autopolyploid species is 0.005%. Nevertheless, such estimates vary between species and may also involve environmental effects [50].

Fourthly, although rare, in addition to diploids, even tetraploids can serve as parental plants where these are common. The triploid hybrid is more likely to be formed from 4x × 3x and 2x × 4x crosses than from a 4x × 4x cross. In these crosses, diploids are not formed, because n = x gametes in tetraploids are extremely rare [50]. Hence, hybridisation can be bidirectional.

Similarly, NEWHYBRIDS categorised the majority of triploid plants to one or another species. Triploid hybrids may arise from all three cytotypes. In contrast to diploids, the tetraploids are mostly admixed and determined as *A. rohlenae* backcrosses. This can be explained by their origins from diploids in the past.

### 4.2. Maintenance of the Hybrid Zone

We supposed a high degree of sterility for the triploid seeds but, surprisingly, found on average that 26.0% of such seeds germinated under the “warm treatment” and 23.2%, under the “cold treatment”. Despite this, the total number of triploid parent trees was very low, and these were found only in the hybrid zone. The question is why the triploids were less frequently found as adult trees. In our samples, they contributed a total of only 3.5%. The maintenance and stability of the hybrid zone is dependent on hybrid fitness, and this is controlled by both intrinsic and extrinsic factors [60]. The strength of intrinsic hybrid selection likely also varies between hybrids over generations, a common observation being that F1 generation hybrids may have higher fitness, while most of the hybrid breakdown occurs in the recombinant generations [61,62]. In the study of Brennan et al. [63], second and third generation hybrids exhibited low relative fitness in terms of seed germination, indicating intensive selection against them. In the tension zone model, the fitness of the F1 generation is not necessarily high but instead may be low, resulting in a limited dispersal from the hybrid to the allopatric populations [1,62]. Additionally, extrinsic factors may play key roles in hybrid selection. Thus, it is known that young seedlings of *Alnus* species are especially susceptible to fungal diseases (e.g., *Phytophtora alni* ssp. *alni*), as are the young of many plant species [64,65]. We speculate that if the triploid hybrids have reduced fitness, it could be that they are less resistant to diseases, so most of them will perish soon after germination. Roe et al. [60] found a selection against hybrids in the hybrid zone of *Populus balsamifera* and *P*. *deltoides* in Canada. The germination rate of the viable seeds was the same as, or even higher than, that of the parental species. On the other hand, the hybrids had lower resistance to several fungal diseases (e.g., *Melampsora* sp.) and died soon after germination.

We assume the vitality of the F1 hybrids is highly variable as is also, thus, that of their descendants, this being influenced by various factors responsible for the disappearance of young hybrid plants from the stands. This may explain the low incidence of adult triploid hybrids and support the persistence of the tension zone.

### 4.3. Differences in Germination

The germination of the tetraploids and diploids varied from 37.9% to 47.5%. The relatively high proportion (over 50%) of empty seeds is quite a common feature of tree seeds in general and of *Alnus glutinosa* s. str. in particular [66]. The differences in germination depended on the species. However, some of the populations within one species germinated well while some of them germinated very poorly or not at all (especially the triploids). Temperature influenced only the rate of germination with a “warm treatment” preference. Gosling et al. [26] set their trial temperatures up to even 35 °C and used seeds from trees collected in England. The temperatures suitable for germination were very wide-ranging, ranging from 10 to 35 °C. Only the duration of the prechill period limited germination. Therefore, it is not entirely clear whether temperature is the limiting factor for germination or if other environmental factors (e.g., rainfall, periods of drought, soil type, etc.) play roles in species distribution. Ashcroft et al. [67] found that variables such as the summer maximum temperature do not play important roles in species distribution, whereas soil conditions and winter minimum temperatures were found to be environmental factors that limited species. The geology and pedology of the southwest Balkan Peninsula are different from those of eastern and northern parts. Limestone and marble are common in the area of the tetraploids. As for the pedology, the soils in the tetraploid zone are of different types—cambisols and leptosols surrounded by planosols, phaeozems, luvisols and umbrisols, with a predominance of diploids (http://www.europe-geology.eu). Therefore, it would seem most likely there are a number of factors that limit species distribution other than temperature.

### 4.4. Unbalanced Germination of Triploids

In Supplementary Table S3, differences in the germination of triploids are evident even within the same population. However, this result is limited by the low numbers of triploids used in our germination experiment (only four individuals, from two populations). The fruits were collected during the same period and at the same stage of maturity, but the differences in germination between samples 226g1228 and 226g1240 are especially significant. The reason for this may be related to how the triploid plants were formed. The four most probable types of pollination that form the triploids are 2n × 4n, 4n × 2n, 3n × 4n and 4n × 3n. A number of studies have shown it is very important if the diploid pollinated the tetraploid plant (2n × 4n) or if the tetraploid pollinated the diploid plant (4n × 2n). Yang et al. [68] found that in mulberries (*Morus* sp.), pollination in both directions can form a proportion of triploid offspring but that the seeds from a 4n × 2n cross are much less likely to germinate. The opposite pattern was found in *Citrus* species [59,69,70]; the germination percentage of seeds from a 4n × 2n cross was much higher than that from a 2n × 4n cross. This may be due to a disturbance of meiosis, with an unequal segregation of chromosomes in the gametes, that results in a greatly reduced fertility of the adult F1 hybrids [71]. Whether the germination was reduced in the first or second example, the triploid progeny that germinate from such seeds are likely to themselves produce sterile or mostly sterile seeds, or their germination percentage will be highly variable [72,73,74]. These considerations may shed the light on our germination findings and explain the wide fluctuations in triploid germination. Unfortunately, we do not know in which way the triploid mother plant was formed, nor do we know which the pollinator of the hybrid was. Since both individuals were assigned to the diploid groups by the SSR and cpDNA analyses, we suppose they were most likely formed by the fusion of an unreduced 2n and reduced 2n gamete or by 2n × 4n and 2n × 3n crosses. Clearly, further research is needed to determine how the sterility or fertility of the triploid hybrids occurs.

### 4.5. Type and Origin of Hybrid Zone

A hybrid zone can be established in a number of ways. We consider two of these. The first, and least likely, is as a consequence of primary intergradation, resulting from a direct response to an environmental gradient [75,76]. The second, and more likely, is of a secondary origin caused by postglacial warming and species expansion [77]. During glaciation, the tetraploids probably remained near coastal areas and in the river deep gorges while the diploids more likely survived in the lowlands north of the Dinaric Alps. The hybrid zone could then have arisen either recently, after the end of the Last Ice Age, or earlier, during the Last Interglacial period (approx. 115,000 years ago). The low number of triploids (3.5%) and their distribution within the hybrid zone may indicate the type of hybrid zone in this case. The STRUCTURE results clearly separated the pure tetraploid from the pure diploid populations with no (or minimal) admixture. Meanwhile, in the vicinity of the hybrid zone, the finding was the other way around. A similar result is evident regarding haplotype sharing. Whereas the area of diploid occurrence on the Balkan Peninsula is well known [17], we can assume that the hybrid zone is narrow and roughly circular in shape, surrounding the entire tetraploid area. From our results, we suggest that there is gene flow between both species and that the low presence of triploids points to some level of selection against them. Therefore, we assume it most likely that a tension zone model prevails, where hybridisation persists as parents move into the hybrid zone, while introgression is limited (but not completely suppressed) by negative selection against hybrids. This allows species to maintain their overall genetic integrity [1,62,71,78,79]. However, we know already that some triploids may have been formed by the fusion of reduced and unreduced gametes, and therefore, these exist outside the hybrid zone between the diploids and tetraploids [17]. The tension zone is the second most common type of hybrid zone described for many plant species (e.g., [80,81]) and also for animal taxa (e.g., [82,83]).

## 5. Conclusions

By combining molecular analyses, a germination experiment and the determination of the ploidy of germinated seeds, we were able to explain gene transfer between two species and the important role played by fertile triploids. Despite the relatively low presence of hybrids, their function is essential because they increase the overall diversity in the region and give rise to new genetic lineages. On the other hand, the processes described significantly complicate species determination in the field if one is working in an area where a hybrid zone has been established.

## Figures and Tables

**Table 1:** Results of the ANOVAs comparing the effects of the temperature treatments (Regime) and *Alnus* taxa on the final germination percentage and germination rate (*t50*).

Final Germination	Df	*F*-Value	*p*-Value
Taxa	2	17.879	<0.001 ***
Regime	1	2.824	0.094
Taxa × Regime	2	0.147	0.864
**Germination rate**			
Taxa	2	5.217	0.006
Regime	1	18.982	<0.001 ***
Taxa × Regime	2	1.308	0.272

*** (*p* < 0.001).

## Supplementary Materials

The following are available online at https://www.mdpi.com/2073-4425/11/7/770/s1. Table S1: Locality details of *Alnus* accessions sampled; Table S2: Differences in germination between *Alnus glutinosa* s. str., *A. rohlenae* and triploids in both climatic regimes; Table S3: General overview of germinated triploid seeds.
