# Supplementary material for: Analyses of Hybrid Viability across a Hybrid Zone between Two Alnus Species Using Microsatellites and cpDNA Markers
Source: Genes (Basel). 2020 Jul 9;11(7):770. doi: 10.3390/genes11070770 (PMC7397206; doi:10.3390/genes11070770)
Supplement: Supplementary file 1 [file genes-11-00770-s001.zip › Supplementary Figure.docx]

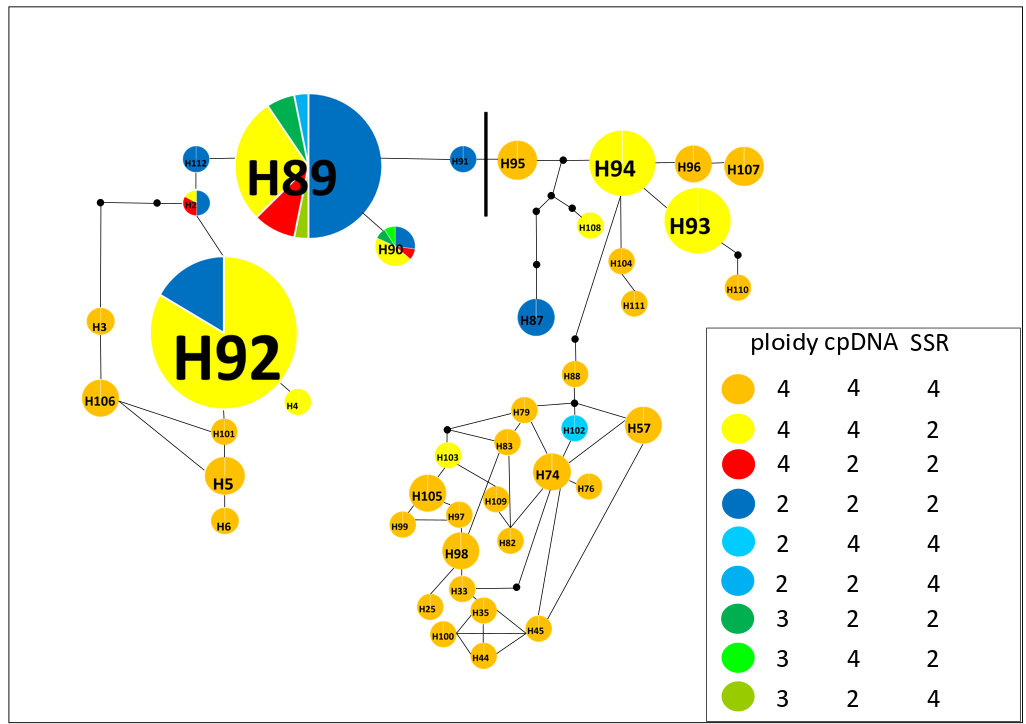


**Supplementary Figure 1.** Haplotype network performed in TCS program. Pie charts represents all nine combinations of ploidy, cpDNA assignment and SSR assignment discovered across the hybrid zone. If one of the three analyzes (estimation of ploidy, haplotype and STRUCTURE analysis) was not performed, the individual was not included in the pie chart. Black line between haplotypes H91 and H95 divide Haplogroup I. from Haplogroup II. as it can be seen in Figure 2. Size of the pie chart depends on the number of individuals inside.
