# Supplementary material for: Analyses of Hybrid Viability across a Hybrid Zone between Two Alnus Species Using Microsatellites and cpDNA Markers
Source: Genes (Basel). 2020 Jul 9;11(7):770. doi: 10.3390/genes11070770 (PMC7397206; doi:10.3390/genes11070770)
Supplement: Supplementary file 1 [file genes-11-00770-s001.zip › Supplementary Tables.docx]

**Supplementary Table S1.** Locality details of sampled *Alnus* accessions. Pop – identification number of populations; N1 – number of individuals used for flow cytometry; N2 – number of individuals used for cpDNA analysis; N3 – number of individuals used for SSR analysis; * populations used for *g*ermination experiment; *A* – average number of alleles; *He* – genetic diversity.

| Pop | N1 | N2 | N3 | *A* | *He* | Haplotype | Ploidy | Latitude | Longitude | Country |
| --- | --- | --- | --- | --- | --- | --- | --- | --- | --- | --- |
| 224 | 3 | 2 | 2 | 4.81 | 0.7 | H87 | 2× | 42.9003 | 21.6844 | SRB |
| 225 | 3 | 1 | 1 | 4.79 | 0.71 | H88 | 2×, 4× | 43.2302 | 21.5300 | SRB |
| 226* | 20 | 14 | 20 | 4.86 | 0.71 | H89, H90, H91 | 2×, 3×, 4× | 43.0475 | 21.3294 | SRB |
| 227* | 20 | 18 | 20 | 4.69 | 0.73 | H89, H92, H93, H94, H2 | 3×, 4× | 42.9891 | 21.3336 | SRB |
| 228* | 18 | 14 | 16 | 4.7 | 0.72 | H89, H90, H92, H93 | 2×, 4× | 43.4487 | 21.2390 | SRB |
| 229* | 18 | 8 | 14 | 4.55 | 0.71 | H2, H89, H94 | 2×, 4× | 43.3367 | 21.2052 | SRB |
| 230 | 3 | 1 | 3 | 4.62 | 0.67 | H89 | 4× | 43.3464 | 21.1180 | SRB |
| 231 | 3 | 1 | 2 | 4.92 | 0.84 | H92 | 4× | 43.3696 | 20.9964 | SRB |
| 232 | 3 | 2 | 2 | 4.81 | 0.8 | H92, H93 | 4× | 43.303 | 20.8953 | SRB |
| 233 | 3 | 0 | 3 | 4.61 | 0.69 | – | 4× | 43.2553 | 20.6630 | SRB |
| 234 | 3 | 2 | 2 | 4.72 | 0.74 | H90, H93 | 4× | 43.3517 | 20.5653 | SRB |
| 235 | 3 | 2 | 3 | 4.87 | 0.73 | H95 | 4× | 43.3979 | 20.7378 | SRB |
| 236 | 3 | 3 | 3 | 4.98 | 0.68 | H92, H93 | 4× | 43.3886 | 20.7670 | SRB |
| 237 | 3 | 2 | 3 | 5.02 | 0.7 | H92, H93 | 4× | 43.4727 | 20.6892 | SRB |
| 238 | 3 | 3 | 3 | 4.57 | 0.69 | H96 | 4× | 43.4788 | 20.5407 | SRB |
| 239 | 3 | 3 | 3 | 4.76 | 0.71 | H79, H92, H97 | 4× | 43.6115 | 20.5529 | SRB |
| 240 | 3 | 3 | 3 | 4.93 | 0.78 | H98 | 4× | 43.6543 | 20.5449 | SRB |
| 241 | 5 | 4 | 5 | 4.91 | 0.71 | H89, H90 | 4× | 43.5904 | 20.9495 | SRB |
| 242 | 3 | 2 | 3 | 4.79 | 0.68 | H89 | 4× | 43.6547 | 20.9958 | SRB |
| 243* | 11 | 7 | 11 | 4.74 | 0.72 | H2, H35, H94, H101, H102 | 2×, 3×, 4× | 43.2090 | 21.6606 | SRB |
| 244* | 17 | 11 | 15 | 4.79 | 0.71 | H2, H44, H90, H93, H94, H100, H103, H104 | 4× | 43.227 | 21.5148 | SRB |
| 245* | 21 | 18 | 18 | 4.84 | 0.72 | H45, H90, H93, H95, H105, H106, H107, H109, H110 | 4× | 43.2142 | 21.3882 | SRB |
| 246* | 11 | 7 | 7 | 4.85 | 0.71 | H90, H93, H111 | 2×, 3×, 4× | 43.1316 | 21.3575 | SRB |
| 247* | 17 | 11 | 15 | 4.65 | 0.71 | H93, H95, H104, H105, H106, H112 | 4× | 43.2023 | 21.2835 | SRB |
| 248* | 15 | 13 | 13 | 4.78 | 0.72 | H89 | 2× | 43.3445 | 21.3255 | SRB |
| 249* | 17 | 13 | 14 | 4.77 | 0.71 | H92 | 4× | 43.3413 | 21.1599 | SRB |
| 252 | 3 | – | – | – | – | – | 2× | 43.3459 | 21.6408 | SRB |
| 204 | 17 | 15 | – | – | – | H3, H4, H5, H6, H25, H57, H74, H82, H83 | 4× | 43.7651 | 20.6652 | SRB |
| 205 | 17 | 15 | – | – | – | H2 | 2× | 43.0344 | 21.6624 | SRB |
| 206 | 18 | 17 | – | – | – | H33, H74, H76 | 4× | 42.8831 | 20.7950 | RKS |
| Total | 287 | 234 | 204 | 4.78 | 0.72 |  |  |  |  |  |

**Supplementary Table S2.** Differences in germination between *Alnus glutinosa* s. str., *A*. *rohlenae* and triploids in both climatic regimes. Means ± Standard Errors (SE) are shown. Those bearing the same letter were not significantly different between levels of particular treatments in multiple-range comparisons (Tukey's test; *P* < 0.05).

| Species | Regime | Germination (%) ± SE |
| --- | --- | --- |
| *A. glutinosa* s. str. | Warm | 47.5±4.5a |
|  | Cold | 44.6±2.5a |
| *A. rohlenae* | Warm | 43.2±5.1a |
|  | Cold | 36.9±5.0a |
| Triploid | Warm | 32.0±3.2c |
|  | Cold | 26.4±2.8c |

**Supplementary Table S3.** General overview of germinated triploid seeds. N1 – total amount of seeds; N2 – number of germinated seeds; % – percentage of germinated seeds.

| Triploid plant | Regime | N1 | N2 | % |
| --- | --- | --- | --- | --- |
| 226g1228 | 1 | 60 | 43 | 71.7 |
| 226g1228 | 2 | 60 | 43 | 71.7 |
| 226g1240 | 1 | 80 | 1 | 1.3 |
| 226g1240 | 2 | 80 | 0 | 0.0 |
| 246g1201 | 1 | 80 | 4 | 5.0 |
| 246g1201 | 2 | 80 | 7 | 8.8 |
| 246g1202 | 1 | 60 | 30 | 50. 0 |
| 246g1202 | 2 | 60 | 15 | 25.0 |
